# The association between blood biological age at rehabilitation admission and physical activity during rehabilitation in geriatric inpatients: RESORT

**DOI:** 10.1007/s11357-024-01152-w

**Published:** 2024-04-09

**Authors:** Jessica K. Lu, Lihuan Guan, Weilan Wang, Anna G. M. Rojer, Fedor Galkin, Jorming Goh, Andrea B. Maier

**Affiliations:** 1 Centre for Healthy Longevity, National University Health System, Singapore, Singapore; 2 Healthy Longevity Translational Research Program, @Age Singapore, Yong Loo Lin School of Medicine, National University of Singapore, Singapore, Singapore; 3 Department of Human Movement Sciences, @AgeAmsterdam, Faculty of Behavioural and Movement Sciences, Vrije Universiteit Amsterdam, Amsterdam Movement Sciences, Van Der Boechorstsraat 7, 1081 BT Amsterdam, The Netherlands; 4 Deep Longevity, Hong Kong, China; 5 Department of Physiology, Yong Loo Lin School of Medicine, National University of Singapore, Singapore, Singapore

**Keywords:** Ageing, Sedentary behaviour, Physical activity, Rehabilitation, Accelerometry

## Abstract

Geriatric rehabilitation inpatients have high levels of sedentary behaviour (SB) and low levels of physical activity (PA). Biological age predicted by blood biomarkers is indicative of adverse outcomes. The objective was to determine the association between blood biological age at rehabilitation admission and levels of SB and PA during rehabilitation in geriatric inpatients. Inpatients admitted to geriatric rehabilitation wards at the Royal Melbourne Hospital (Melbourne, Australia) from October 22, 2019, to March 29, 2020, in the REStORing health of acute unwell adulTs (RESORT) observational cohort were included. Blood biological age was predicted using SenoClock-BloodAge, a hematological ageing clock. Patients wore an inertial sensor to measure SB and PA. Logistic regression analyses were conducted. A total of 111 patients (57.7% female) with mean age 83.3 ± 7.5 years were included in the analysis. The mean blood biological age was 82.7 ± 8.4 years. Patients with 1-year higher blood biological age had higher odds of having high SB measured as non-upright time greater than 23 h/day (odds ratio (OR): 1.050, 95% confidence interval (CI): 1.000–1.102). Individuals having 1-year higher age deviation trended towards lower odds of having high levels of PA measured as stepping time greater than 7.4 min/day (OR: 0.916, CI: 0.836–1.005) and as greater than 19.5 sit-to-stand transitions/day (OR: 0.915, CI: 0.836–1.002). In conclusion, higher biological age was associated with higher levels of SB and trended towards lower PA. Incorporating blood biological age could facilitate resource allocation and the development of more tailored rehabilitation plans.

**Supplementary Information:**

The online version contains supplementary material available at 10.1007/s11357-024-01152-w.

## Introduction

An individual’s biological age is a measure of the level of biological functioning of the body compared to an expected level of functioning for a corresponding chronological age [1]. This measurement can be obtained using fast-changing biomarkers, such as blood creatinine levels and blood cell counts, by integrating them as biological ageing clocks [2]. Fast-changing biomarkers respond rapidly to changes in the body after an acute event. Thus, the ageing clocks may be useful for predicting recovery and adverse adaptations [3]. During hospitalization, individual biomarkers, such as albumin and C-reactive protein, can predict physical function [4], and composite biomarkers, such as the frailty index based on blood parameters, can predict mortality [5]. Similarly, higher biological age predicted by blood biomarkers denotes accelerated ageing rates, which is indicative of mortality [6, 7] and a set of health conditions [8] in community-dwelling and hospitalized populations.

Geriatric rehabilitation represents a post-acute care setting to enhance residual functional capacity [9], particularly in older adults experiencing functional decline [10]. These inpatients usually have high levels of sedentary behaviour (SB) and low levels of physical activity (PA) [11, 12]. In community-dwelling older adults, higher SB and lower PA are associated with negative outcomes such as falls, impaired activities of daily living, cognitive impairment, and mortality [13]. Higher inactivity in hospitalized older adults can also lead to adverse consequences, such as physical and cognitive decline [14], and poses challenges to recovery in rehabilitation programs. Earlier identification of patients with potential inactivity using blood biological age may enhance rehabilitation planning and resource allocation. Application of biological ageing clocks in a geriatric rehabilitation inpatient population has been less studied; thus, the association between blood biological age and the levels of SB and PA is not well understood.

This study aimed to determine the association between blood biological age at rehabilitation admission and the levels of SB and PA during rehabilitation in geriatric inpatients.

## Materials and methods

### Study design

REStORing health of acutely unwell adulTs (RESORT) is an observational, longitudinal, and prospective cohort of geriatric rehabilitation inpatients admitted to the Royal Park Campus of the Royal Melbourne Hospital (Melbourne, Australia). More details on this cohort are presented in prior publications [12, 15, 16]. Briefly, after acute hospitalization, patients who required comprehensive care to restore functional capacity were transferred to geriatric rehabilitation wards. A Comprehensive Geriatric Assessment (CGA) was used to assess physical, psychological, functional, nutrition, and sociological domains within 48 h of rehabilitation admission. Written informed consent was obtained from inpatients or nominated proxies. Patients were excluded if they were unable to provide informed consents, without a legal proxy to consent, or undergoing palliative care at admission. Inpatients from the RESORT cohort without bilateral lower extremity paralysis were eligible for inclusion in the Ending PyJama (PJ) Paralysis campaign [11], and no further restrictions on ambulation status were present. As part of this campaign, a random sample of inpatients from two out of four geriatric rehabilitation wards wore an inertial sensor (ActivPal4, PAL Technologies Ltd, Glasgow, Scotland, UK) to measure instrumented SB and PA from October 22, 2019, to March 29, 2020. There were 145 patients who wore the ActivPal4. This study was approved by the Melbourne Health Human Research Ethics Committee (HREC/17/MH/103) with all ethical guidelines adhered to in accordance with the Declaration of Helsinki [17].

### Data collection

Age, sex, ethnicity, education, and length of stay in geriatric rehabilitation were retrieved from medical records. A stadiometer was used to assess standing height if the inpatient could stand. Otherwise, knee height was assessed, from which height was calculated [18]. Weight was assessed using a standing scale, seated scale, or a weighted hoist depending on the patient’s ambulation status. Body mass index (BMI) was calculated using body mass (kg) divided by height (m) squared and expressed in kg/m^2^. The primary reason for hospital admission was categorized into cardiovascular, musculoskeletal, neurological, psychiatry, respiratory, and other reasons. Principal diagnoses, including falls, fractures, and functional decline, were extracted from medical records.

Comorbidity was assessed using the Cumulative Illness Rating Scale (CIRS, range 0–56) with higher scores indicating greater comorbidity burden, which is calculated by dividing the total score with the number of affected physiological systems [19]. The usage of medication was obtained from medical records. Frailty was assessed by the Clinical Frailty Scale (CFS, range 0–9) with higher scores indicating increased frailty and associated risks [20]. Cognitive impairment was defined as a dementia diagnosis reported in medical records, standard Mini-Mental State Examination (sMMSE) score < 24/30 [21], Montreal Cognitive Assessment (MoCA) score < 26/30 [22], and/or Rowland Universal Dementia Assessment Scale (RUDAS) score < 23/30 [23], if further cognitive testing was indicated. The Short Confusion Assessment Method was used to assess the risk of delirium [24]. The Hospital Anxiety and Depression Scale (range 0‒21) was used to assess significant anxiety and depression symptoms with a cut-off score of ≥ 8 [25]. The use of a walking aid and history of falls were self-reported by patients and/or carers. The Functional Ambulation Classification (FAC, range 0‒5) was used for assessing ambulation status with higher scores indicating less support is required (i.e. independent) [26]. Handgrip strength was measured using a handheld dynamometer (JAMAR hand dynamometer; Samsons Preston, Inc.) on both hands three times each, alternating each time [27]. The maximum value (kg) was used for analyses. Inpatients who were unable to perform handgrip strength tests due to medical reasons were ascribed 0.00 m/s or 0.0 kg. The Short Physical Performance Battery (SPPB, range 0–12) was used to assess physical performance with higher scores indicating better performance [28]. The Katz index of activities of daily living (KADL, range 0–6) [29] and the Lawton and Brody scale of instrumental ADL (IADL, range 0–8) [30] were used to measure functional performance with higher scores indicating greater living independency. Malnutrition risk was assessed by the Malnutrition Screening Tool (MST) by which patients with a score ≥ 2 were classified as at risk [31].

### Blood biological age prediction

Biological age using blood parameters was predicted using the BloodAge clock, available via the online SenoClock platform (https://www.deeplongevity.com/senoclock) developed by Deep Longevity, Hong Kong (subsidiary of Regent Pacific 00575.HK) [2]. SenoClock-BloodAge is a modular ensemble of 21 deep neural networks (DNNs) trained using over 60,000 samples from common blood biochemistry and cell count tests. A total of 30 clinical frequently measured blood biochemistry and cell count parameters were input in the biological age prediction: albumin, hemoglobin, white blood cells, platelets, hematocrit, red blood cell, mean corpuscular volume, mean corpuscular hemoglobin, mean corpuscular hemoglobin concentration, red cell distribution width, mean platelet volume, neutrophils, lymphocytes, monocytes, eosinophils, basophils, sodium, potassium, chloride, calcium, phosphorous, blood urea nitrogen, creatinine, total protein, total globulin, total bilirubin, alanine transaminase, aspartate transaminase, gamma-GT, and alkaline phosphatase. Blood tests for the abovementioned parameters undertaken close to rehabilitation admission after acute care were included. If one blood parameter was unavailable, the patient was excluded from the analysis.

### Objective measurement of sedentary behaviour and physical activity

Details on the collection of physical activity data is presented elsewhere [11, 12, 15]. In brief, from day 5 (range 3–7) after rehabilitation admission, patients wore an ActivPAL4 inertial sensor on their right thigh for 7 days, or until hospital discharge, to objectively assess daily SB and PA. The ActivPAL4 consists of a triaxial capacitive accelerometer with a range of ± 4 g that collected data in 15-s epochs at a sampling frequency of 20 Hz and analyzed in 60-s epochs. A valid day of measurements was defined as 20 out of 24 h of wear. Patients were included in the analyses if they reported at least one valid day. The ActivPAL software (Generation 8, PAL Technologies Ltd.) was used to generate eight SB and PA measures, which were averaged over valid days [11, 15]. Daily objectively measured SB patterns were described by the median of the mean time spent non-upright (sum of sitting and lying), mean sitting time, and mean lying time in hours/day. Daily objectively measured PA patterns were described by the median of the mean time spent upright (sum of standing and stepping), mean standing time, and mean stepping time in minutes/day, and the median of the mean number of steps and mean sit-to-stand (STS) transitions per day.

### Statistical analyses

Descriptive statistics for continuous variables with a normal distribution were presented as means ± standard deviations (SD) and a non-normal distribution as medians [interquartile ranges, IQR]. Categorical variables were presented as numbers (percentages). Numerical variables were compared using independent sample *t*-tests (normal distribution) and the Mann–Whitney *U* tests (skewed distribution), and categorical variables were compared using ${\chi }^{2}$-tests or Fisher’s exact tests (categorical variables).

The independent variables were blood biological age and age deviation, for which age deviation was defined as the difference between blood biological age and chronological age (i.e. blood biological age minus chronological age). A positive difference indicates an individual is biologically older than their chronological age. The dependent variables, the eight SB and PA measures, were dichotomized using the median as a cut-off into groups of patients with low/high SB and low/high PA. The association of blood biological age or age deviation with objectively measured SB and PA measures was investigated using binary logistic regression analyses. Analyses included a crude model and a model adjusted for CIRS. Results are presented as odds ratios (OR) with 95% confidence intervals (CI).

The statistical significance level was set at $\alpha \mathrm{ = 0.05}$, and a trend was defined as an $\alpha$ value of greater than 0.05 and less than 0.10. Analyses were performed using the IBM SPSS Statistics for Macintosh, Version 27.0 (IBM Corp.).

## Results

### Patient characteristics

Out of 145 patients with available physical activity data, 111 patients with complete blood biochemistry data were included in the analysis. No clinically significant differences were observed between the 111 included and 34 excluded patients (Supplementary Material, Table 1). The characteristics of the included patients (57.7% female) with a mean age of 83.3 ± 7.5 years are summarized in Table 1. The mean blood biological age of the patients was 82.7 ± 8.4 years. The median length of stay in geriatric rehabilitation was 18.0 [IQR: 11.9–32.7] days. The median BMI was 26.9 [22.9–31.5] kg/m^2^. The most common primary reason for hospital admission was musculoskeletal (49.5%). The median CIRS and CFS scores were 12 [8-16] and 6 [5–7], respectively. The median FAC score was 2 [1–3], and the KADL and IADL scores were 2 [1–3] and 1 [0–2], respectively. In the past year, 76.6% of patients had a fall and 67.9% of patients use a walking aid.

**Table 1:** Characteristics of inpatients at admission to geriatric rehabilitation

	*n*	Total (*N* = 111)
Age, years	111	83.3 ± 7.5
Blood biological age, years	111	82.7 ± 8.4
Female, *n* (%)	111	64 (57.7)
European/Caucasian, *n* (%)	111	95 (86.4)
Education, years	83	9.0 [6.0–11.0]
Length of stay in rehabilitation, days	111	18.0 [11.9–32.7]
BMI, kg/m^2^	107	26.9 [22.9–31.5]
**Primary reasons for hospital admission, *n* (%)**	111	
Musculoskeletal		55 (49.5)
Neurological		15 (13.5)
Respiratory		8 (7.2)
Psychiatry		7 (6.3)
Cardiac		6 (5.4)
Other		20 (18.0)
**Principal diagnoses, *n* (%)**	111	
Fall		30 (27.0)
Fracture		22 (19.8)
Functional decline		21 (18.9)
**Morbidity and frailty**		
CIRS score [0–56], points	111	12 [8–16]
CIRS severity index, points	111	2.0 ± 0.5
Number of medications	111	8.8 ± 4.8
CFS score [0–9], points	100	6 [5–7]
**Cognition and psychology**		
Cognitive impairment, *n* (%)	111	72 (64.9)
Delirium, *n* (%)	111	21 (18.9)
Anxiety (HADS score ≥ 8), *n* (%)	88	40 (45.5)
Depression (HADS score ≥ 8), *n* (%)	86	44 (51.1)
**Physical function and nutrition**		
Use of a walking aid, *n* (%)	111	76 (68.5)
Fall in the past year, *n* (%)	109	82 (77.1)
FAC score [0–5], points	106	2 [1–3]
Handgrip strength, kg	91	
Female	52	13.0 ± 6.9
Male	39	22.7 ± 7.3
SPPB score [0–12], points	103	1 [0–4]
KADL score [0–8], points	111	2 [1–3]
IADL score [0–6], points	111	1 [0–2]
At risk of malnutrition (MST score ≥ 2), *n* (%)	107	39 (36.4)
**Objectively measured physical activity**		
Wearing time, days	111	6 [6–6]
Non-upright time, hours/day	111	23.1 [22.0–23.6]
Sitting time	111	8.8 [2.4–11.6]
Lying time	111	12.8 [9.9–20.5]
Upright time, minutes/day	111	55.0 [26.3–120.7]
Standing time	111	44.4 [23.6–102.3]
Stepping time	111	7.4 [1.4–14.6]
Steps, number/day	111	417 [64.9–910.0]
Sit-to-Stand transitions, number/day	111	19.5 [9.3–30.0]

Data is presented as mean ± standard deviation (SD) or median [interquartile range (IQR)] unless otherwise stated

*BMI*, body mass index; *CFS*, Clinical Frailty Scale; *CIRS*, Cumulative Illness Rating Scale; *FAC*, Functional Ambulation Classification; *HADS*, Hospital Anxiety and Depression Scale; *IADL*, Instrumental Activities of Daily Living; *IQR*, interquartile ranges; *KADL*, Katz Index of Activities of Daily Living; *kg*, kilogram; *MST*, Malnutrition Screening Tool; *SD*, standard deviation; *SPPB*, Short Physical Performance Battery

### Objectively measured sedentary behaviour and physical activity measures

The ActivPAL4 was worn for a median wearing duration of 6 [6–6] days. The median non-upright time was 23.1 [22.0–23.6] hours/day (h/d), of which the median sitting time was 8.8 [2.4–11.6] h/d and median lying time was 12.8 [9.9–20.5] h/d. The median upright time was 55.0 [26.3–120.7] minutes/day (mins/d), of which median standing time was 44.4 [23.6–102.3] mins/d and median stepping time was 7.4 [1.4–14.6] mins/d. The median step count was 417 [64.9–910.0] steps/day, and the median number of STS transitions was 19.5 [9.3–30.0] per day (Table 1).

### Association of blood biological age with objectively measured sedentary behaviour and physical activity

**Fig. 1:**
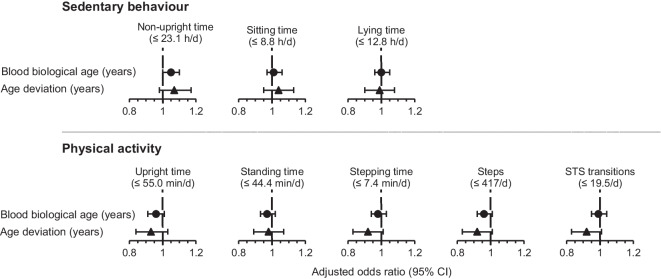
The association between blood biological age or age deviation and objectively measured sedentary behaviour (SB) and physical activity (PA) in geriatric rehabilitation inpatients (*n* = 111). The reference group is patients with low SB measured as non-upright time (≤ 23.1 h/d), sitting time (≤ 8.8 h/d), and lying time (≤ 12.8 h/d) or low PA measured as upright time (≤ 55.0 min/d), standing time (≤ 44.4 min/d), stepping time (≤ 7.4 min/d), steps (≤ 417/d), and sit-to-stand (STS) transitions (≤ 19.5/d). CI, 95% confidence interval; d, day; h, hours; min, minutes

The association between blood biological age and the odds of having high SB and high PA are shown in Fig. 1. For every 1 year higher in blood biological age, patients had higher odds of having high SB measured as non-upright time greater than 23.1 h/d (OR: 1.052, 95% CI: 1.003–1.102, $\mathit{p}=0.037$) (Table 2) and a trend towards lower odds of having high PA measured as upright time greater than 55.0 min/d (OR: 0.957, 95% CI: 0.914–1.003, $\mathit{p}=0.067$) (Table 3). In the comorbidity adjusted model, for every 1 year higher in blood biological age, patients had higher odds of having high SB measured as non-upright time greater than 23.1 h/d (OR: 1.050, 95% CI: 1.000–1.102, *p* = 0.048) (Table 2) and a trend towards lower odds of having high PA measured as upright time greater than 55.0 min/d (OR: 0.959, 95% CI: 0.914–1.006, *p* = 0.087) (Table 3).

**Table 2:** The association between blood biological age or age deviation and objectively measured sedentary behaviour measures in geriatric rehabilitation inpatients using binary logistic regression analysis (*N* = 111)

	Non-upright time > 23.1 h/d			Sitting time > 8.8 h/d			Lying time > 12.8 h/d		
	OR	95% CI	*P*	OR	95% CI	*P*	OR	95% CI	*P*
**Blood biological age (years)**									
Crude	1.052	1.003–1.102	**0.037**	1.012	0.968–1.059	0.595	0.999	0.956–1.045	0.970
Adjusted model	1.050	1.000–1.102	**0.048**	1.011	0.966–1.057	0.642	1.000	0.956–1.046	0.987
**Age deviation (years)**									
Crude	1.074	0.982–1.174	0.118	1.038	0.951–1.134	0.401	0.985	0.902–1.075	0.731
Adjusted model	1.070	0.976–1.173	0.148	1.035	0.948–1.131	0.442	0.987	0.904–1.078	0.776

Age deviation: blood biological age subtracted by chronological age

Interpretation: One year higher in blood biological age or age deviation is associated with higher/lower odds of higher sedentary behaviour measures when compared to lower sedentary behaviour measures

Adjusted model: comorbidity (CIRS) adjusted

*P* < 0.050 presented in bold

*CI*, confidence interval; *d*, day; *h*, hours; *OR*, odds ratio

**Table 3:** The association between blood biological age or age deviation and objectively measured physical activity measures in geriatric rehabilitation inpatients using binary logistic regression analysis (*N* = 111)

	Upright time > 55.0 min/d			Standing time > 44.4 min/d			Stepping time > 7.4 min/d			Steps > 417/d			Sit-to-stand transitions > 19.5/d		
	OR	95% CI	*P*	OR	95% CI	*P*	OR	95% CI	*P*	OR	95% CI	*P*	OR	95% CI	*P*
**Blood biological age (years)**															
Crude	0.957	0.914–1.003	0.067	0.968	0.925–1.013	0.166	0.977	0.934–1.022	0.315	0.963	0.920–1.009	0.111	0.986	0.943–1.031	0.545
Adjusted model	0.959	0.914–1.006	0.087	0.970	0.926–1.016	0.198	0.980	0.935–1.026	0.379	0.965	0.921–1.011	0.134	0.987	0.944–1.033	0.572
**Age deviation (years)**															
Crude	0.927	0.848–1.014	0.098	0.971	0.889–1.060	0.507	0.914	0.835–1.000	0.051	0.915	0.836–1.002	0.055	0.914	0.835–1.001	0.051
Adjusted model	0.930	0.849–1.020	0.124	0.975	0.892–1.066	0.584	0.916	0.836–1.005	0.064	0.918	0.838–1.006	0.067	0.915	0.836–1.002	0.056

Age deviation: blood biological age subtracted by chronological age

Interpretation: One year higher in blood biological age or age deviation is associated with higher/lower odds of higher physical activity measures when compared to lower physical activity measures

Adjusted model: comorbidity (CIRS) adjusted

*CI*, confidence interval; *d*, day; *min*, minutes; *OR*, odds ratio

### Association of age deviation and objectively measured sedentary behaviour and physical activity

The association between age deviation and the odds of having high SB and high PA are shown in Fig. 1. No statistically significant association was observed between age deviation and measures of SB (Table 2). For every 1 year higher in age deviation, patients trended towards lower odds of having high PA measured as upright time greater than 55.0 min/d (OR: 0.927, 95% CI: 0.848–1.014, ${\mathrm{p}}\mathrm{=0.098}$), stepping time greater than 7.4 min/d (OR: 0.914, 95% CI: 0.835–1.000, ${\mathrm{p}}\mathrm{=0.051}$), step count greater than 417 steps/day (OR: 0.915, 95% CI: 0.836–1.002, ${\mathrm{p}}\mathrm{=0.055}$), and STS transitions greater than 19.5 per day (OR: 0.914, 95% CI: 0.835–1.001, ${\mathrm{p}}\mathrm{=0.051}$) (Table 3). In the comorbidity adjusted model, for every 1 year higher in age deviation, patients trended towards lower odds of having high PA measured as stepping time greater than 7.4 min/d (OR: 0.916, 95% CI: 0.836–1.005, ${\mathrm{p}}\mathrm{=0.064}$), step count greater than 417 steps/day (OR: 0.918, 95% CI: 0.838–1.006, ${\mathrm{p}}\mathrm{=0.067}$), and STS transitions greater than 19.5 per day (OR: 0.915, 95% CI: 0.836–1.002, ${\mathrm{p}}\mathrm{=0.056}$).

## Discussion

Higher blood age was significantly associated with high SB measured as higher non-upright time and trended towards having low PA measured as lower upright time. Being biologically older than chronological age (i.e. higher age deviation) was not significantly associated with the levels of SB. However, it trended towards having low PA measured as lower upright time, stepping time, steps, and STS transitions.

Physical activity measures have been progressively introduced into clinical settings as a vital sign indicating the general physical condition and an individual’s physical function [32]. Physical function refers to the capacity of an individual to execute the physical activities of daily living and reflects motor function and control, physical fitness, and habitual bodily movement [33]. This is an important health indicator, especially for adults 65 years and older, as acute hospitalization usually results in loss of function in basic ADLs for up to 50% of patients during hospitalization [34]. In the present study, patients were physically inactive for most of the day, with high levels of SB and low levels of PA. Even with higher nursing staff availability at the bedside, to encourage and assist in PA, differences were minimal and having more staff did not affect SB and PA [11]. Thus, this indicates that those who are bedbound are more likely to have low PA while those with high PA measurements were likely to not be bedbound and have better physical function. A similar circumstance was observed in nursing-home residents where those with poorer health status have limited mobility [35].

In the present study, a higher blood biological age was associated with high SB and a trend of low PA. The ageing clock technology is currently being used in various products for health screening and is at the foundation of the emerging longevity medicine field [36, 37]. Additionally, ageing clocks are widely used in anti-ageing research and are actively being patented for commercial applications [38, 39]. Ageing clocks that rely on clinical blood tests are especially well-fit for the needs of longevity medicine due to their compatibility with the existing logistics and practices of the healthcare industry.

A prior study in these patients showed that worse morbidity, malnutrition, and poor physical and functional performance as well as depressive symptoms were associated with higher SB and lower PA [15]. Moreover, two previously published blood-based ageing clocks showed associations between blood biological age and morbidity: higher DNAm PhenoAge is associated with higher comorbidities in adults aged 21–100 years from the United States [40] and older biological age was associated with more comorbidity in the Berlin Ageing Study (BASE) and BASE II in older adults aged 70–103 years [41]. Thus, comorbidity was theorized as a potential confounder as worse morbidity is a determinant of PA [15] and certain diseases induce changes in blood biomarkers; however, the results showed that the association of blood biological age with levels of SB and PA was independent of comorbidity. Higher biological age may be associated with high SB and a trend of low PA because a higher biological age indicates poorer health status [6, 7], which contributes to limitations in well-being and reduced capacity to be more active [42, 43]. Additionally, psychological factors, such as unhappiness or loneliness, can increase blood biological age [8]. Psychological factors, such as distress and well-being, are associated with limitations in daily activities, hindering motivation to move or to be more active and engage in activities [44, 45].

This is the first study exploring the association of biological age and levels of (in)activity in hospitalized older adults. The RESORT cohort stands out encompassing a diverse population [46, 47], including older adults undergoing general rehabilitation whereas other studies may include only those receiving post-acute care for conditions such as stroke [48] and cardiac complications [49]. Another strength of this study includes using the SenoClock-BloodAge DNN ageing clock, which is responsive and can potentially reflect a patient’s dynamic health condition, compared to epigenetic clocks like DNAm PhenoAge [40] and GrimAge [50]. Moreover, PhenoAge and GrimAge use DNA methylation patterns to predict biological age instead of using clinical blood biomarkers; thus, SenoClock-BloodAge confers higher ease of assessment and lower costs as blood test results could be retrieved from medical records directly without needs for DNA extraction and epigenetic profiling. A limitation of this study is as SenoClock-BloodAge was trained in healthy community-dwelling individuals across a wide age range, which includes fewer older adults, the DNN may have limitations in estimating the biological age of a population that is much older with multimorbidity. However, the DNN could measure a patient’s pace of ageing for predicting morality due to COVID-19 [7], suggesting promising applications of SenoClock-BloodAge across other clinical settings. Additionally, a larger sample size would increase the statistical power to explore the association between blood biological age and the levels of SB and PA. More insights into the connection between ageing and activity may be gained by comparing the biological age of patients between rehabilitation admission and hospital discharge as an increase in mobility due to geriatric rehabilitation is predicted to translate into ageing deceleration, where the rate of biological ageing slows down. Such a study would help validate deep biomarkers of ageing as an indicator or a predictor of clinical response in the current geriatric rehabilitation inpatient population, which has been less studied using biological ageing clocks.

In conclusion, higher blood biological age was associated with prolonged non-upright time and a trend of shorter upright time in geriatric rehabilitation inpatients. Patients who were biologically older tended to have low levels of PA. This biological age estimation based on blood biomarkers could be used when tailoring rehabilitation plans to potentially identify patients who are more likely to be inactive and require more resources to improve their PA. Nevertheless, future studies with a larger sample size are required to confirm the relationship of blood biological age with levels of SB and PA and more functional outcomes.

### Supplementary Information

Below is the link to the electronic supplementary material.

Supplementary file1 (DOCX 34 KB)

## Data Availability

All data generated or analyzed during this study are included in this article. Further enquiries can be directed to the corresponding author.
