# Supplementary material for: The association between blood biological age at rehabilitation admission and physical activity during rehabilitation in geriatric inpatients: RESORT
Source: GeroScience. 2024 Apr 9;46(5):4505–15. doi: 10.1007/s11357-024-01152-w (PMC11336003; doi:10.1007/s11357-024-01152-w)
Supplement: Supplementary file 1 — Supplementary file1 (DOCX 34 KB) [file 11357_2024_1152_MOESM1_ESM.docx]

# Supplementary Material

Title:

**The association between blood biological age at rehabilitation admission and physical activity during rehabilitation in geriatric inpatients: RESORT**

Jessica K. Lu, MEng^1,2^, Lihuan Guan, PhD^1,2^, Weilan Wang, PhD^1,2^, Anna G.M. Rojer, MD, PhD^3^, Fedor Galkin, MSc^4^, Jorming Goh, PhD^1,2,5^, Andrea B. Maier, MD, PhD^1,2,3^ ​

^1^ Centre for Healthy Longevity, National University Health System, Singapore, Singapore.

^2^ Healthy Longevity Translational Research Program, Yong Loo Lin School of Medicine, National University of Singapore, Singapore, Singapore.

^3^ Department of Human Movement Sciences, @AgeAmsterdam, Faculty of Behavioural and Movement Sciences, Vrije Universiteit Amsterdam, Amsterdam Movement Sciences, the Netherlands.

^4^ Deep Longevity, Hong Kong, China.

^5^ Department of Physiology, Yong Loo Lin School of Medicine, National University of Singapore, Singapore, Singapore.

Correspondence to: Prof Dr Dr Andrea B Maier, @Age, Department of Human Movement Sciences, Faculty of Behavioural and Movement Sciences, Vrije Universiteit Amsterdam, Amsterdam Movement Sciences, Van der Boechorstsraat 7, 1081 BT Amsterdam, the Netherlands

Email: a.b.maier@vu.nl

# Table 1. Comparison of the characteristics of inpatients with available physical activity data at admission to geriatric rehabilitation stratified by completeness of blood biochemistry data.

|  | **n** | **Complete (*N* = 111)** | **n** | **Incomplete (*N* = 34)** | ***P*** |
| --- | --- | --- | --- | --- | --- |
| Age, years | 111 | 83.3 ± 7.5 | 34 | 82.0 ± 8.6 | 0.411 |
| Female, n (%) | 111 | 64 (57.7) | 34 | 17 (50.0) | 0.431 |
| European/Caucasian, n (%) | 111 | 95 (86.4) | 34 | 31 (91.2) | 0.732 |
| Education, years | 83 | 9.0 [6.0–11.0] | 29 | 10.0 [8.0–13.5] | 0.051 |
| Length of stay in rehabilitation, days | 111 | 18.0 [11.9–32.7] | 34 | 13.4 [9.06–20.1] | 0.054 |
| BMI, kg/m^2^ | 107 | 26.9 [22.9–31.5] | 34 | 28.0 [24.3–31.7] | 0.243 |
| **Primary reasons for hospital admission, n (%)** | 111 |  | 34 |  | 0.671 |
| Musculoskeletal |  | 55 (49.5) |  | 16 (47.1) |  |
| Neurological |  | 15 (13.5) |  | 5 (14.7) |  |
| Respiratory |  | 8 (7.2) |  | 4 (11.8) |  |
| Psychiatry |  | 7 (6.3) |  | 2 (5.9) |  |
| Cardiac |  | 6 (5.4) |  | 3 (8.8) |  |
| Other |  | 20 (18.0) |  | 4 (11.7) |  |
| **Principal diagnoses, n (%)** | 111 |  | 34 |  |  |
| Fall |  | 30 (27.0) |  | 8 (23.5) | 0.685 |
| Fracture |  | 22 (19.8) |  | 10 (29.4) | 0.238 |
| Functional decline |  | 21 (18.9) |  | 5 (14.7) | 0.575 |
| **Morbidity and frailty** |  |  |  |  |  |
| CIRS score [0–56], points | 111 | 12 [8–16] | 34 | 12 [9–17] | 0.340 |
| CIRS severity index, points | 111 | 2.0 ± 0.5 | 34 | 2.2 ± 0.5 | 0.087 |
| Number of medications | 111 | 8.8 ± 4.8 | 34 | 10.6 ± 4.2 | 0.064 |
| CFS score [0–9], points | 100 | 6 [5–7] | 29 | 6 [5–7] | 0.698 |
| **Cognition and psychology** |  |  |  |  |  |
| Cognitive impairment, n (%) | 111 | 72 (64.9) | 34 | 17 (50.0) | 0.119 |
| Delirium, n (%) | 111 | 21 (18.9) | 34 | 6 (17.6) | 0.868 |
| Anxiety (HADS score ≥ 8), n (%) | 88 | 40 (45.5) | 22 | 8 (36.3) | 0.662 |
| Depression (HADS score ≥ 8), n (%) | 86 | 44 (51.1) | 21 | 12 (57.1) | **0.006** |
| **Physical function and nutrition** |  |  |  |  |  |
| Use of a walking aid, n (%) | 111 | 76 (68.5) | 33 | 23 (69.7) | 0.894 |
| Fall in the past year, n (%) | 109 | 84 (77.1) | 34 | 24 (70.6) | 0.443 |
| FAC score [0–5], points | 106 | 2 [1–3] | 34 | 3 [1–3] | 0.242 |
| Handgrip strength, kg | 91 |  | 31 |  |  |
| Female | 52 | 13.0 ± 6.9 | 15 | 15.7 ± 4.7 | 0.175 |
| Male | 39 | 22.7 ± 7.3 | 16 | 20.3 ± 6.9 | 0.269 |
| SPPB score [0–12], points | 103 | 1 [0–4] | 33 | 2 [0–5] | 0.700 |
| KADL score [0–8], points | 111 | 2 [1–3] | 34 | 2 [1–3] | 0.388 |
| IADL score [0–6], points | 111 | 1 [0–2] | 34 | 1 [0–2] | 0.223 |
| At risk of malnutrition (MST score ≥ 2), n (%) | 107 | 39 (36.4) | 34 | 11 (32.4) | 0.664 |
| **Objectively measured physical activity** |  |  |  |  |  |
| Wearing time, days | 111 | 6 [6–6] | 34 | 6 [4–6] | **0.022** |
| Non-upright time, hours/day | 111 | 23.1 [22.0–23.6] | 34 | 23.0 [22.0–23.6] | 0.907 |
| Sitting time | 111 | 8.8 [2.4–11.6] | 34 | 9.6 [2.6–11.5] | 0.826 |
| Lying time | 111 | 12.8 [9.9–20.5] | 34 | 12.9 [10.4–19.6] | 0.929 |
| Upright time, minutes/day | 111 | 55.0 [26.3–120.7] | 34 | 62.3 [23.2–121.6] | 0.959 |
| Standing time | 111 | 44.4 [23.6–102.3] | 34 | 48.7 [19.2–96.4] | 0.794 |
| Stepping time | 111 | 7.4 [1.4–14.6] | 34 | 7.0 [1.5–15.2] | 0.707 |
| Steps, number/day | 111 | 417 [64.9–910.0] | 34 | 388 [70.5–977.8] | 0.721 |
| Sit-to-Stand transitions, number/day | 111 | 19.5 [9.3–30.0] | 34 | 21.6 [9.4–30.6] | 0.716 |

Data is presented as mean ± standard deviation (SD) or median [interquartile range (IQR)] unless otherwise stated.

Abbreviations: BMI, Body Mass Index; CFS, Clinical Frailty Scale; CIRS, Cumulative Illness Rating Scale; FAC, Functional Ambulation Classification; HADS, Hospital Anxiety and Depression Scale; IADL, Instrumental Activities of Daily Living; IQR, interquartile ranges; KADL, Katz Index of Activities of Daily Living; kg, kilogram; MST, Malnutrition Screening Tool; SD, standard deviation; SPPB, Short Physical Performance Battery. Patients with complete blood biochemistry data were included in the logistic regression analyses and those with incomplete blood biochemistry data were excluded from the logistic regression analyses.
